# Medication Compliance in Schizophrenia: The Role of Sociodemographic Factors and Depression

**DOI:** 10.1192/j.eurpsy.2025.2202

**Published:** 2025-08-26

**Authors:** S. Ben Aissa, R. Khouloud, N. Chaima, L. Amine, M. Wahid

**Affiliations:** 1 Psychiatry D, Razi Hospital, Mannouba, Tunisia

## Abstract

**Introduction:**

Non-adherence to prescribed medications in schizophrenia is a primary factor leading to relapse and repeated hospitalizations, posing a significant challenge in psychiatric care. Understanding and enhancing medication adherence is crucial for improving outcomes in schizophrenia treatment.

**Objectives:**

This study aimed to estimate the prevalence of medication non-adherence among schizophrenia patients and to explore the impact of depression and other sociodemographic factors on adherence levels. Recognizing these influences is essential for developing targeted interventions to improve patient outcomes.

**Methods:**

A cross-sectional analysis was conducted involving 350 individuals with schizophrenia, encompassing both outpatients and inpatients. A comprehensive questionnaire was employed to collect data on sociodemographic characteristics, clinical history, and therapeutic interventions.

To assess medication adherence and depressive symptoms, the study utilized the Medication Adherence Rating Scale (MARS) and the Calgary Depression Scale for Schizophrenia (CDSS), both of which are validated tools designed for psychiatric evaluations.

**Results:**

Data analysis revealed that 21.3% of the participants exhibited poor medication adherence as measured by MARS. Further statistical testing using a logistic regression model identified employment status and level of depression as significant predictors of non-adherence.

Patients who were unemployed and those exhibiting higher scores on the CDSS indicating more severe depressive symptoms were more likely to be non-compliant with their medication regimen (p=0,001). This highlights the intricate relationship between mental health symptoms and treatment adherence.

**Conclusions:**

The results of this study illuminate the importance of considering depression and employment status when addressing medication adherence in individuals with schizophrenia. These factors play a pivotal role in the adherence behavior and overall treatment outcomes of patients.

Emphasizing comprehensive care that includes management of depression alongside routine antipsychotic treatment could enhance adherence and improve the prognosis for individuals living with schizophrenia. Ongoing research is needed to further elucidate the pathways that influence medication adherence and to develop strategies that effectively address these challenges.

**Disclosure of Interest:**

None Declared

